# Nonlinear and Age‐Specific Associations Between Atherogenic Index of Plasma and Differential Cardiovascular Risk Profiles in US Adults

**DOI:** 10.1155/cdr/6691842

**Published:** 2026-01-21

**Authors:** Yuelin Hu, Xuli Chen, Yanchao Liu, Qiuyu Wang, Wenwen Xiao

**Affiliations:** ^1^ Department of Electrocardiology, The Second Affiliated Hospital of Wannan Medical College, Wuhu, China, wnmc.edu.cn; ^2^ Department of Health Education, Eastern Theater Command Centers for Disease Control and Prevention, Nanjing, China

**Keywords:** age interaction, atherogenic index of plasma, cardiovascular disease, NHANES, threshold effect

## Abstract

**Background:**

The association between the atherogenic index of plasma (AIP) and different cardiovascular disease (CVD) outcomes remains insufficiently studied.

**Methods:**

Data from 16,302 US adults in NHANES 2005–2020 were analyzed. CVD outcomes included congestive heart failure, coronary heart disease, angina, heart attack, and stroke. Multivariable logistic regression models assessed the linear association between AIP and CVD. Restricted cubic splines were used to examine potential nonlinear relationships, whereas threshold effect analysis identified inflection points. Stratified analyses explored interactions between AIP and covariates.

**Results:**

Compared with the lowest quartile, the highest AIP quartile was associated with increased odds of overall CVD (OR = 1.73, 95% CI = 1.20–2.52, *p* for trend = 0.001), coronary heart disease (OR = 2.20, 95% CI = 1.20–4.25, *p* for trend = 0.013), and heart attack (OR = 2.53, 95% CI = 1.42–4.74, *p* for trend = 0.008). A U‐shaped relationship was observed between AIP and congestive heart failure (*p* overall = 0.0004, *p* nonlinear = 0.002), with a sharp odds increase when AIP exceeded 0.22 (OR = 3.40, 95% CI = 1.81–6.38, *p* < 0.01). Subgroup analysis revealed a significant age interaction: Among adults younger than 55 years, each AIP increment was associated with higher odds of CVD (OR = 2.53, 95% CI = 1.60–3.98, *p* = 0.001), heart attack (OR = 2.58, 95% CI = 1.21–5.54, *p* = 0.014), and stroke (OR = 2.98, 95% CI = 1.44–6.17, *p* = 0.003).

**Conclusion:**

Elevated AIP is an independent predictor of CVD, coronary heart disease, and heart attack, particularly in younger adults (< 55 years). A U‐shaped relationship was identified between AIP and congestive heart failure. These findings highlight AIP as a valuable biomarker for CVD risk assessment and emphasize the importance of age‐specific intervention strategies targeting different CVD outcomes.

## 1. Introduction

Globally, disorders of the circulatory system constitute the foremost etiology of premature death and loss of healthy life years, with contemporary epidemiological studies attributing nearly one‐third of all mortality to cardiovascular etiologies [1, 2]. The cardiovascular disease (CVD) spectrum comprises diverse clinical manifestations such as ischemic heart disease, cerebrovascular accidents, and peripheral vascular insufficiency, predominantly stemming from atherosclerotic vascular remodeling—a complex immunopathological phenomenon involving lipid deposition and chronic vascular wall inflammation [3]. The progression of CVD often leads to myocardial infarction, heart failure, and other severe complications, imposing a tremendous burden on healthcare systems and reducing patients′ quality of life [4]. Despite notable advances in prevention and treatment, the global prevalence of CVD continues to escalate, particularly in low‐ and middle‐income countries, highlighting the need for improved risk prediction and early intervention strategies.

The development of CVD is influenced by a variety of modifiable and nonmodifiable risk factors, including gender, age, lifestyle, medical history, obesity, and genetic predisposition [5–7]. Among these factors, lipid metabolism disorders play a central role in initiating and accelerating atherosclerosis and contributing to CVD pathophysiology [8, 9]. Dyslipidemia, characterized by elevated levels of triglycerides (TGs), low‐density lipoprotein cholesterol (LDL‐C), and reduced high‐density lipoprotein cholesterol (HDL‐C), is closely associated with endothelial dysfunction, oxidative stress, and vascular inflammation [10, 11]. Abnormal lipid profiles contribute to the formation of small, dense LDL particles, which are more prone to oxidation and can easily penetrate the arterial endothelium, accelerating the progression of atherosclerosis [12]. Given the central role of lipid metabolism in the development of CVD, identifying reliable lipid‐related biomarkers for risk assessment has become a critical focus in cardiovascular research.

The atherogenic index of plasma (AIP) has emerged as an innovative metric for evaluating dyslipidemia patterns and CVD susceptibility [13]. It quantifies the equilibrium between pro‐atherogenic and anti‐atherogenic lipid fractions and serves as a proxy for estimating the size and density of low‐density lipoprotein (LDL) particles. Higher AIP values denote an increased prevalence of small, dense LDL particles, which exhibit greater atherogenic potential and significantly elevate the risk of CVD [14]. Extensive epidemiological evidence has consistently linked elevated AIP levels with increased CVD‐specific mortality [15]. Moreover, AIP has been shown to outperform traditional lipid parameters in predicting CVD risk and has been linked to metabolic syndrome, insulin resistance, and other cardiometabolic disorders [16, 17]. However, despite its promising potential as a predictor of cardiovascular events, the relationship between AIP and CVD risk remains insufficiently characterized across diverse populations, underscoring the need for further investigation to establish its clinical utility.

To bridge this knowledge gap, our study leveraged data from the National Health and Nutrition Examination Survey (NHANES), spanning 2005–2020, to comprehensively assess the association between AIP and CVD outcomes. Using multivariable regression models, we evaluated the dose–response relationship between AIP and CVD risk while accounting for a wide range of demographic, lifestyle, and clinical covariates. Moreover, we conducted stratified analyses to explore potential effect modifications across subgroups, such as age, sex, and comorbidities. By utilizing a nationally representative, population‐based dataset, our investigation provides robust evidence to delineate the impact of AIP on CVD risk and highlights its potential as a biomarker for refining risk stratification and guiding targeted intervention strategies. These findings contribute meaningfully to advancing our understanding of AIP′s role in CVD pathophysiology and offer valuable insights for mitigating the global burden of CVD.

## 2. Methods

### 2.1. Study Population

The NHANES, overseen by the National Center for Health Statistics under the Centers for Disease Control and Prevention, is a comprehensive, cross‐sectional program designed to assess the health status and nutritional habits of the US population. This survey provides a nationally representative sample of the noninstitutionalized US population, enabling the evaluation of chronic disease risk factors and health trends over time.

**Figure 1:**
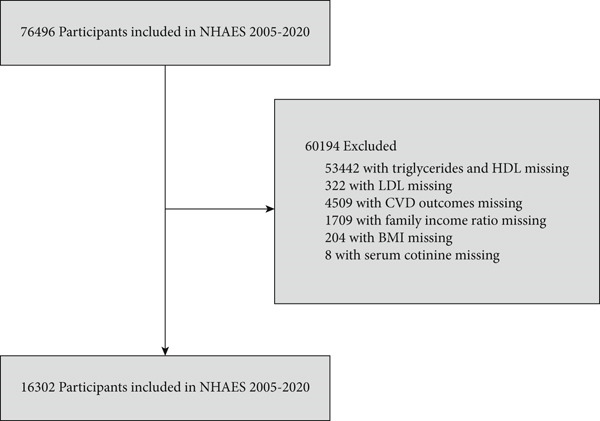
Flowchart of inclusion and exclusion in the study.

Our study utilized NHANES data spanning 2005–2020, with an initial sample comprising 76,496 participants. Rigorous exclusion criteria were subsequently applied, resulting in the removal of 60,194 participants due to the following reasons: A total of 53,442 participants were excluded due to missing data on TG and HDL. A total of 322 participants had incomplete data on LDL. A total of 4,509 participants lacked information on CVD‐related outcomes. A total of 1,709 participants had missing family income ratio data. A total of 204 participants had no recorded body mass index (BMI) data. A total of 8 participants were missing serum cotinine measurements. After applying these exclusions, a total of 16,302 participants were retained for analysis, comprising 14,544 non‐CVD participants and 1,758 participants with CVD. The detailed inclusion and exclusion process is visually summarized in Figure 1.

### 2.2. Measurement and Calculation of AIP

In this study, the AIP was the variable of interest, calculated as the ratio of TGs to HDL‐C. AIP provides valuable information for assessing lipid metabolism and CVD risk. TG levels were quantified using an enzymatic hydrolysis method. In this method, TGs undergo hydrolysis by lipase, resulting in the formation of glycerol and free fatty acids. The generated glycerol undergoes subsequent oxidation, producing hydrogen peroxide, which reacts with a chromogenic indicator in the presence of peroxidase, leading to a measurable color change. The intensity of this color change, assessed through spectrophotometry, is directly proportional to the TG concentration. HDL was measured using either a direct assay method or a selective inhibition method, where HDL interacts with specific reagents in the reaction system to produce a colored product, which is quantified using spectrophotometry.

### 2.3. Outcomes

CVD outcomes were defined by participants′ self‐report of “having ever been told” by a clinician that they had the disease, and that these measures therefore represent prevalent (lifetime) disease history rather than incident events. The outcomes variable of this study was overall CVD and its subtypes, obtained through a specialized medical questionnaire. Participants were asked: “Have you ever been told that you have congestive heart failure (CHF), coronary heart disease, angina, heart attack, or stroke?” If a participant responded “yes” to any of these conditions, they were classified as having CVD.

### 2.4. Covariates

We accounted for multiple covariates as potential confounders, encompassing demographic characteristics, medical history, lifestyle behaviors, and biochemical indicators, which were meticulously selected based on prior literature. Demographic, health, and lifestyle data were obtained through structured interviews conducted by NHANES‐trained personnel. Alcohol consumption status was assessed using the query “Do you have four or more drinks almost every day?” and categorized as yes, no, or unknown. Smoking status was determined by the response to “Have you smoked at least 100 cigarettes in your lifetime?” and classified as yes, no, or unknown. BMI was calculated as weight (kg) divided by height squared (m^2^). Biochemical measurements, including serum cotinine, TG, HDL, and LDL‐C, were derived from blood samples collected in a controlled environment and analyzed at certified laboratories adhering to quality control protocols.

### 2.5. Statistical Analysis

We summarized the baseline characteristics of participants by analyzing the distribution of covariates. Continuous variables were expressed as means with standard deviations (mean ± SD). Categorical variables were displayed as frequencies and percentages. Independent *t*‐tests were utilized to compare continuous variables across groups, whereas categorical data were analyzed using chi‐square tests to detect differences between groups. Our research objective was to estimate the exposure–outcome association (adjusted odds ratios [ORs] between AIP and various CVD outcomes), rather than directly modeling the overall prevalence. Therefore, this study used unweighted multivariate logistic regression as the main analytical method. To investigate the association between AIP and CVD outcomes, we stratified overall CVD risk and five specific CVD subtypes into quartiles and applied multivariable logistic regression models to estimate adjusted ORs and 95% confidence intervals (CIs), accounting for potential confounders. Model 1 was adjusted for key demographic variables. Model 2 was extended to include additional covariates such as medical history (hypertension and diabetes), lifestyle factors (smoking and alcohol consumption), and physiological markers (BMI, LDL‐C, and serum cotinine). To evaluate potential nonlinear associations between AIP and CVD outcomes, we incorporated restricted cubic splines (RCSs) with three knots, allowing for flexible modeling of dose–response relationships. For significant nonlinear relationships, the inflection point was determined by maximizing the likelihood across potential threshold values. Moreover, stratified analyses were conducted to examine potential effect modifications by key covariates, including age (< 55, ≥ 55), gender, alcohol use, smoking status, hypertension, diabetes, and BMI (< 30, ≥ 30). Interaction terms were tested to explore heterogeneity across subgroups. All statistical analyses were performed using R software (Version 4.4.1), and statistical significance was defined as a *p* value < 0.05.

## 3. Results

### 3.1. Characteristics of the Participants

The characteristics of the study population are summarized in Table 1. A total of 16,302 participants were included, comprising 14,544 non‐CVD individuals and 1,758 CVD patients. Participants with CVD were substantially older than those without CVD (mean age 65.97 ± 12.85 vs. 47.45 ± 17.13 years). The CVD group had a greater proportion of males and a higher representation of non‐Hispanic White participants. Individuals with CVD had higher prevalences of cigarette smoking, alcohol use, diabetes, and hypertension than those without CVD. Additionally, CVD patients exhibited a higher BMI (30.54 ± 7.43) and a greater prevalence of obesity (BMI ≥ 30) compared with non‐CVD individuals (29.03 ± 7.03). Serum cotinine levels were also significantly elevated in the CVD group (69.71 ± 149.86).

**Table 1:** Basic characteristics of the study population.

**Characteristics**	**Overall**	**Non-CVD**	**CVD**	**p** **value**
*n*	16302	14544	1758	
Age (mean [SD])	49.45 (17.68)	47.45 (17.13)	65.97 (12.85)	< 0.001
Gender (%)				< 0.001
Male	7809 (47.9)	6832 (47.0)	977 (55.6)	
Female	8493 (52.1)	7712 (53.0)	781 (44.4)	
Race (%)				< 0.001
Mexican American	2408 (14.8)	2264 (15.6)	144 (8.2)	
Other Hispanic	1540 (9.4)	1407 (9.7)	133 (7.6)	
Non‐Hispanic White	7073 (43.4)	6105 (42.0)	968 (55.1)	
Non‐Hispanic Black	3412 (20.9)	3011 (20.7)	401 (22.8)	
Other race	1869 (11.5)	1757 (12.1)	112 (6.4)	
Education (%)				< 0.001
Less than 9th grade	1573 (9.6)	1334 (9.2)	239 (13.6)	
9th–11th grade	2241 (13.7)	1930 (13.3)	311 (17.7)	
High school graduate/GED or equivalent	3688 (22.6)	3244 (22.3)	444 (25.3)	
Some college or AA degree	4853 (29.8)	4372 (30.1)	481 (27.4)	
College graduate or above	3938 (24.2)	3657 (25.1)	281 (16.0)	
Unknown	9 (0.1)	7 (0.0)	2 (0.1)	
Family income ratio (%)				< 0.001
Mean (SD)	2.53 (1.62)	2.57 (1.63)	2.23 (1.49)	
< 1.3	5021 (30.8)	4394 (30.2)	627 (35.7)	
1.3–3.5	6236 (38.3)	5495 (37.8)	741 (42.2)	
≥ 3.5	5045 (30.9)	4655 (32.0)	390 (22.2)	
Alcohol (%)				< 0.001
Yes	2163 (13.3)	1818 (12.5)	345 (19.6)	
No	11002 (67.5)	9907 (68.1)	1095 (62.3)	
Unknown	3137 (19.2)	2819 (19.4)	318 (18.1)	
Smoke (%)				< 0.001
Yes	7260 (44.5)	6209 (42.7)	1051 (59.8)	
No	9030 (55.4)	8323 (57.2)	707 (40.2)	
Unknown	12 (0.1)	12 (0.1)	0 (0.0)	
Diabetes (%)				< 0.001
Yes	2052 (12.6)	1477 (10.2)	575 (32.7)	
No	13871 (85.1)	12757 (87.7)	1114 (63.4)	
Borderline	372 (2.3)	303 (2.1)	69 (3.9)	
Unknown	7 (0.0)	7 (0.0)	0 (0.0)	
Hypertension (%)				< 0.001
Yes	5871 (36.0)	4582 (31.5)	1289 (73.3)	
No	10409 (63.9)	9943 (68.4)	466 (26.5)	
Unknown	22 (0.1)	19 (0.1)	3 (0.2)	
BMI (%)				< 0.001
Mean (SD)	29.19 (7.09)	29.03 (7.03)	30.54 (7.43)	
< 25	4772 (29.3)	4382 (30.1)	390 (22.2)	
25–30	5366 (32.9)	4807 (33.1)	559 (31.8)	
≥ 30	6164 (37.8)	5355 (36.8)	809 (46.0)	
Serum cotinine (mean (SD))	57.18 (128.11)	55.67 (125.15)	69.71 (149.86)	< 0.001

*Note:* Date was presented as mean (SD) or *n* (%). Categorical variables were analyzed using chi‐square tests continuous variables were analyzed using analysis of variance.

Abbreviations: BMI, body mass index; SD, standard deviation.

### 3.2. Association Between AIP and CVD‐Related Diseases

Table 2 reports associations between AIP (modeled as a continuous variable) and prevalent cardiovascular outcomes. In fully adjusted logistic regression models, a one‐unit increase in AIP was associated with higher odds of any CVD (OR = 1.63, 95% CI = 1.29–2.04, *p* < 0.001), CHF (OR = 1.52, 95% CI = 1.04–2.23, *p* = 0.031), coronary heart disease (OR = 1.75, 95% CI = 1.24–2.47, *p* = 0.001), and heart attack (OR = 1.70, 95% CI = 1.22–2.36, *p* = 0.002).

**Table 2:** Multivariate logistic regression between AIP and CVD outcomes.

**Outcomes**	**Model 1**		**Model 2**	
	**OR (95% CI)**	**p** **value**	**OR (95% CI)**	**p** **value**
CVD	2.24 (1.81, 2.78)	< 0.001	1.63 (1.29, 2.04)	< 0.001 ^∗^
Congestive heart failure	2.48 (1.72, 3.57)	< 0.001	1.52 (1.04, 2.23)	0.031 ^∗^
Coronary heart disease	2.23 (1.61, 3.10)	< 0.001	1.75 (1.24, 2.47)	0.001 ^∗^
Angina	2.02 (1.36, 3.00)	0.001	1.35 (0.90, 2.05)	0.151
Heart attack	2.29 (1.67, 3.13)	< 0.001	1.70 (1.22, 2.36)	0.002 ^∗^
Stroke	1.55 (1.12, 2.16)	0.009	1.15 (0.81, 1.63)	0.426

*Note:* The asterisk ( ^∗^) indicates that the difference is significant.

Abbreviations: OR, odds ratio; CI, confidence interval.

We next examined AIP divided into quartiles using two multivariable logistic regression models (Table 3). In the fully adjusted model (Model 2), the ORs (95% CIs) for overall CVD across increasing AIP quartiles were 1.00 (reference), 1.17 (0.86–1.61), 1.30 (0.95–1.80), and 1.73 (1.20–2.52) (*p* for trend = 0.001), indicating a graded association and a significant linear trend. For coronary heart disease the corresponding ORs were 1.00 (reference), 1.64 (0.97–2.98), 1.87 (1.10–3.42), and 2.20 (1.20–4.25) (*p* for trend = 0.013). For myocardial infarction, ORs across quartiles were 1.00 (reference), 1.72 (1.04–3.05), 1.79 (1.07–3.18), and 2.53 (1.42–4.74) (*p* for trend = 0.008). By contrast, we did not observe statistically significant positive trends across AIP quartiles for CHF, angina, or stroke (*p* for trend > 0.05).

**Table 3:** Association between AIP and CVD outcomes across quartiles.

	**CVD**	**Congestive heart failure**	**Coronary heart disease**	**Angina**	**Heart attack**	**Stroke**
Model 1						
Quartile 1	Ref	Ref	Ref	Ref	Ref	Ref
Quartile 2	1.21 (0.90, 1.66)	0.79 (0.51, 1.30)	1.55 (0.92, 2.79)	1.62 (0.86, 3.47)	1.71 (1.04, 3.01)	0.79 (0.54, 1.20)
Quartile 3	1.53 (1.14, 2.09)	1.10 (0.70, 1.80)	1.90 (1.13, 3.41)	2.20 (1.17, 4.71)	1.97 (1.20, 3.46)	0.96 (0.65, 1.47)
Quartile 4	2.53 (1.79, 3.62)	2.23 (1.32, 3.88)	2.88 (1.61, 5.46)	2.01 (0.95, 4.66)	3.51 (2.01, 6.46)	1.48 (0.91, 2.43)
*p* for trend	< 0.001	< 0.001	< 0.001	0.007	< 0.001	0.011
Model 2						
Quartile 1	Ref	Ref	Ref	Ref	Ref	Ref
Quartile 2	1.17 (0.86, 1.61)	0.70 (0.44, 1.16)	1.64 (0.97, 2.98)	1.53 (0.81, 3.31)	1.72 (1.04, 3.05)	0.74 (0.50, 1.13)
Quartile 3	1.30 (0.95, 1.80)	0.80 (0.50, 1.34)	1.87 (1.10, 3.42)	1.81 (0.95, 3.92)	1.79 (1.07, 3.18)	0.81 (0.54, 1.25)
Quartile 4	1.73 (1.20, 2.52)	1.26 (0.73, 2.26)	2.20 (1.20, 4.25)	1.29 (0.59, 3.03)	2.53 (1.42, 4.74)	1.04 (0.63, 1.75)
*p* for trend	0.001 ^∗^	0.054	0.013 ^∗^	0.464	0.008 ^∗^	0.383

*Note:* The asterisk ( ^∗^) indicates that the difference is significant.

Abbreviation: Ref, reference.

### 3.3. Nonlinear Relationship Between AIP and CVD‐Related Diseases

Given evidence of a potential nonlinear association between AIP and CVD outcomes, we fitted adjusted RCS models to characterize the dose–response relationship. The adjusted smoothing spline for CHF is shown in Figure 2 and indicates a statistically significant nonlinear (U‐shaped) association (*p* overall = 0.0004; *p* nonlinear = 0.002). Threshold effect analysis identified the inflection point of AIP for CHF at 0.22 (Table 4). When AIP ≥ 0.22, a pronounced positive association was observed between AIP and CHF (OR = 3.40, 95% CI = 1.81–6.38, *p* < 0.01), suggesting that each unit increase in AIP increased the risk of CHF by approximately 2.4‐fold. Conversely, on the left side of the inflection point (AIP < 0.22), no statistically significant relationship was detected (OR = 0.51, 95% CI = 0.17–1.57, *p* = 0.23).

Figure 2Nonlinear relationship between AIP and CVD outcomes. (a) overall CVD, (b) congestive heart failure, (c) coronary heart disease, (d) angina, (e) heart attack, and (f) stroke.

**Figure:**
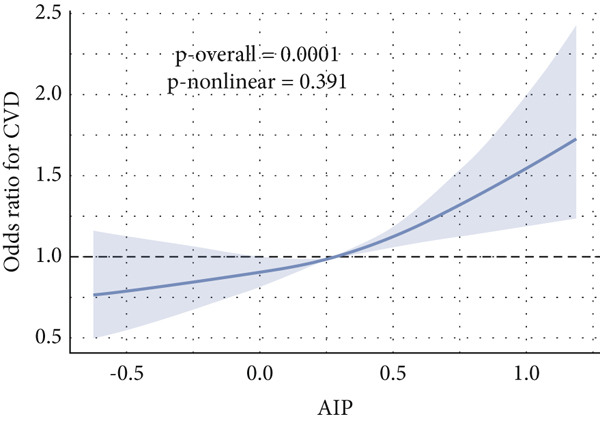
(a)

**Figure:**
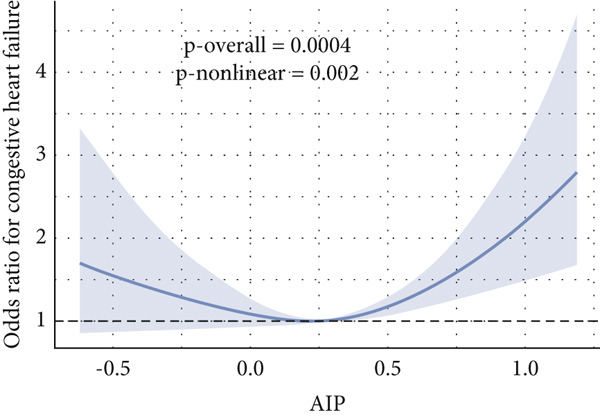
(b)

**Figure:**
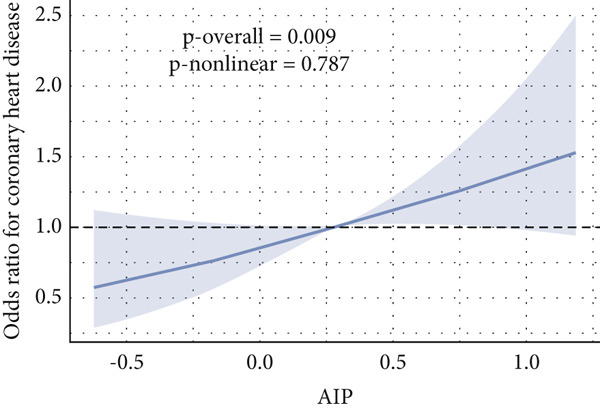
(c)

**Figure:**
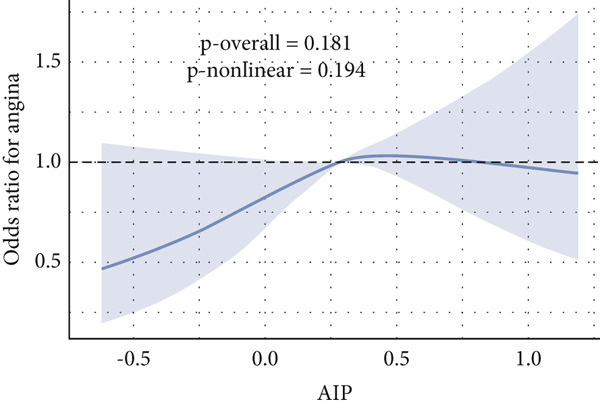
(d)

**Figure:**
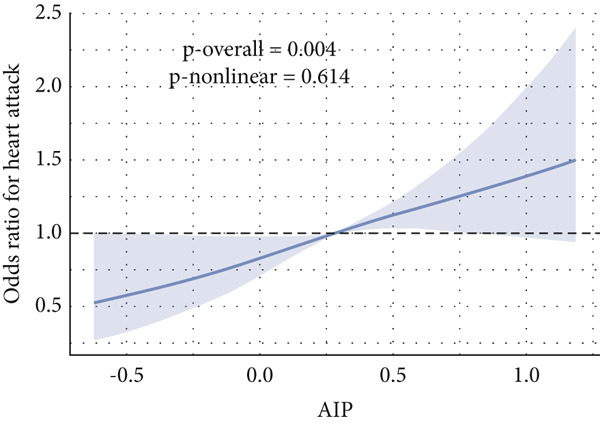
(e)

**Figure:**
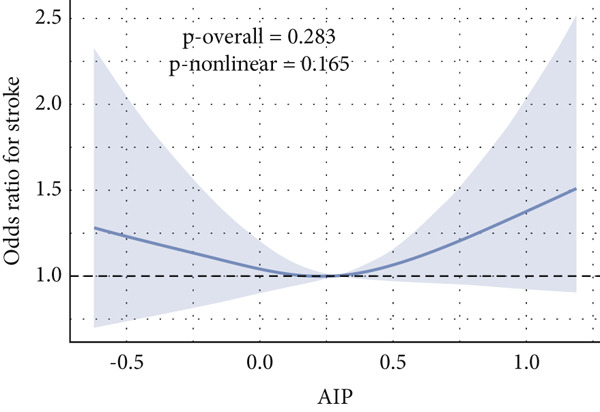
(f)

**Table 4:** Threshold effect analysis of AIP and congestive heart failure.

	**OR**	**95% CI**	**p** **value**
Fitting by linear regression	1.08	0.83–1.4	0.54
Fitting by piecewise binary logistic regression models			
AIP < 0.22	0.51	0.17–1.57	0.23
AIP ≥ 0.22	3.40	1.81–6.38	< 0.01 ^∗^
*p* for log likelihood ratio test			0.21

*Note:* The asterisk ( ^∗^) indicates that the difference is significant.

Abbreviations: CI, confidence interval; OR, odds ratio.

### 3.4. Stratified Analyses

We performed stratified analyses for overall CVD and specific CVD endpoints by age, sex, alcohol consumption, smoking, hypertension, diabetes, and BMI; the detailed results are presented in Figure 3. We found significant interaction effects of age on the associations between AIP and CVD, heart attack, and stroke (*p* interaction < 0.05). In age‐stratified analyses, higher AIP was more strongly associated with prevalent CVD among younger adults. Specifically, in participants aged < 55 years, each unit increase in AIP was associated with markedly higher odds of any CVD (OR = 2.53, 95% CI = 1.60–3.98, *p* = 0.001), myocardial infarction (OR = 2.58, 95% CI = 1.21–5.54, *p* = 0.014), and stroke (OR = 2.98, 95% CI = 1.44–6.17, *p* = 0.003). Among participants aged ≥ 55 years, AIP remained positively associated with any CVD (OR = 1.31, 95% CI = 1.02–1.70, *p* = 0.048) and myocardial infarction (OR = 1.52, 95% CI = 1.06–2.16, *p* = 0.021), but the magnitude of these associations was substantially attenuated compared with the younger group. Notably, in the older stratum (≥ 55 years), AIP showed a nonsignificant inverse association with stroke (OR = 0.83, 95% CI = 0.56–1.22, *p* = 0.336).

Figure 3Stratified analyses of the associations between AIP and CVD outcomes. (a) overall CVD, (b) congestive heart failure, (c) coronary heart disease, (d) angina, (e) heart attack, and (f) stroke.

**Figure:**
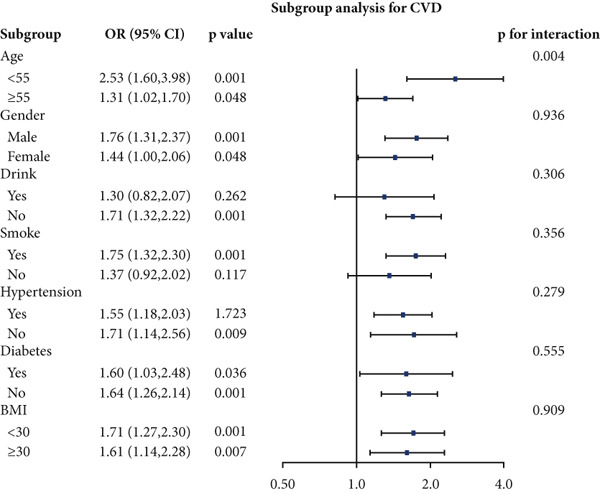
(a)

**Figure:**
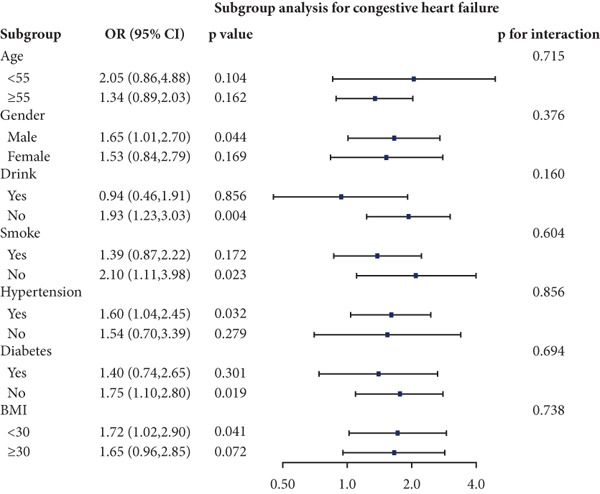
(b)

**Figure:**
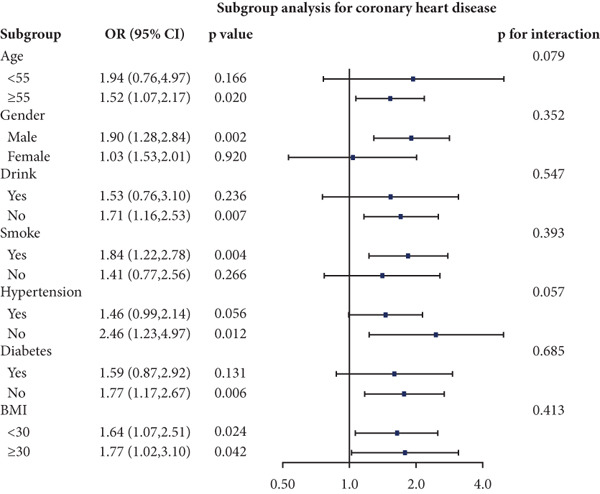
(c)

**Figure:**
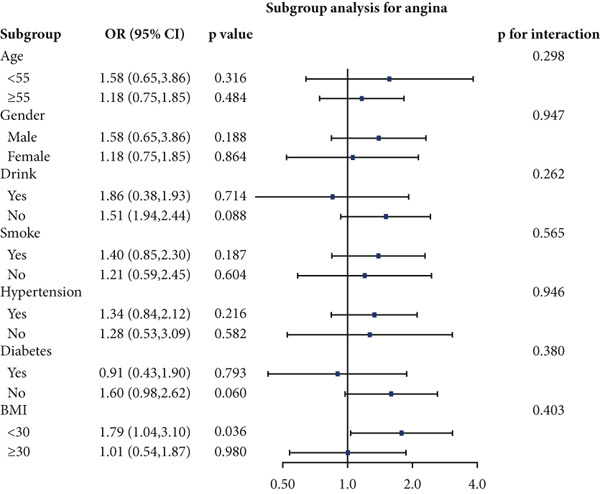
(d)

**Figure:**
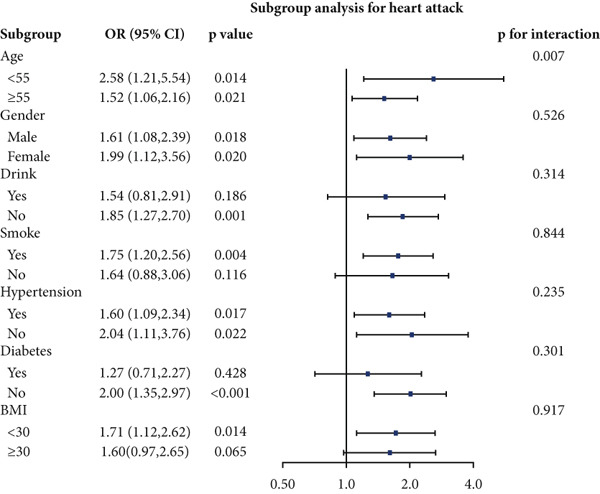
(e)

**Figure:**
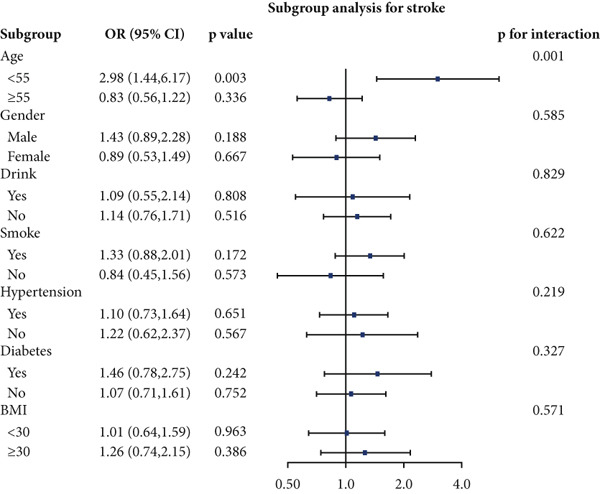
(f)

## 4. Discussion

This study comprehensively examined the relationship between AIP and multiple CVD outcomes. Using data from a nationally representative sample, we applied multivariable logistic regression models to assess the association between AIP and CVD risk. Our findings indicated that elevated AIP levels were significantly correlated with an increased risk of overall CVD, coronary heart disease, and heart attack, demonstrating a consistent upward trend across quartiles (*p* for trend < 0.05). RCS analysis further identified a U‐shaped nonlinear association between AIP and CHF, with a notable elevation in risk when AIP values exceeded 0.22. In addition, stratified analyses revealed that the relationship between AIP and CVD‐related diseases was more pronounced in individuals under 55 years of age, where AIP exhibited stronger associations with CVD, heart attack, and stroke. Conversely, in participants aged ≥ 55 years, the magnitude of risk increase was relatively attenuated. These results underscore the potential role of AIP as an important biomarker for predicting CVD‐related outcomes and suggest that dysregulated lipid metabolism may contribute to CVD pathogenesis through distinct metabolic pathways.

Previous studies have shown that dyslipidemia is a core pathological mechanism of atherosclerotic CVD [18, 19]. The lipid deposition hypothesis, characterized by elevated LDL‐C, along with metabolic disturbances such as dysfunctional HDL‐C and increased TG levels, has been shown to be significantly associated with coronary artery disease and ischemic stroke [20, 21]. However, traditional lipid parameters (such as LDL and HDL) often face limitations in fully capturing the complexity of atherosclerotic lipid metabolism due to their single‐dimensional evaluation (ignoring lipoprotein particle heterogeneity and insulin resistance). Although recent studies have attempted to improve risk prediction using combined indicators such as non‐HDL and the apolipoprotein B/A1 ratio, the AIP serves as a novel biomarker that integrates lipid synthesis and clearance balance [22–24]. However, large‐scale population‐based evidence on the relationship between AIP and CVD remains limited. Our finding that higher AIP is associated with greater odds of prevalent coronary heart disease and self‐reported myocardial infarction is consistent with multiple prior observational studies and recent meta‐analyses showing positive associations between AIP (or the TG/HDL‐C ratio) and coronary artery disease and adverse coronary outcomes. In particular, recent meta‐analytic work and cohort studies have reported that elevated AIP is associated with greater CAD risk, greater disease severity, and poorer prognosis [25]. Evidence relating AIP to stroke and CHF has been more heterogeneous. Some cohort analyses show positive associations between AIP and stroke incidence, whereas a few reports have found weaker or even inverse associations with heart failure prevalence in particular populations [26]. These mixed findings are likely explained by differences in study design (cross‐sectional vs. prospective), endpoint definitions (prevalent self‐report vs. adjudicated incident events), populations (general population vs. selected clinical cohorts), and covariate adjustment.

The biological mechanisms underlying the association between AIP and CVD remain unclear, likely resulting from multiple synergistic mechanisms. AIP is a logarithmic transformation of the TG to HDL‐C ratio and is considered a surrogate marker for atherogenic dyslipidemia and the presence of small, dense LDL particles. Mechanistically, elevated TG and low HDL‐C are linked to increased remnant lipoproteins, impaired reverse cholesterol transport, and greater oxidative and inflammatory activity processes that promote endothelial dysfunction, plaque formation, and plaque instability. AIP also correlates with insulin resistance and other components of the metabolic syndrome, which further accelerate atherogenesis and thrombosis [27, 28]. Elevated AIP suggests a higher proportion of small, dense LDL particles. Studies have shown that elevated LDL particles can more easily penetrate dysfunctional endothelial cells and enter the arterial intima [29]. Within the intima, LDL particles are highly susceptible to oxidative modification by reactive oxygen species, forming oxidized low‐density lipoprotein (oxLDL). This process activates scavenger receptors on endothelial cells and macrophages, triggering inflammatory responses and promoting the formation of foam cells, which constitute the lipid core of atherosclerotic plaques [30, 31]. Additionally, oxLDL contributes to endothelial dysfunction and thrombogenesis. By inhibiting endothelial nitric oxide synthase activity, oxLDL reduces nitric oxide production, leading to impaired vasodilation and disruption of the endothelial barrier [32, 33]. Furthermore, oxLDL upregulates tissue factor (TF) expression and suppresses thrombomodulin, facilitating thrombin generation and promoting platelet aggregation, which ultimately results in the formation of occlusive thrombi [34]. LDL also interacts synergistically with other lipoproteins, such as lipoprotein (a), accelerating the progression of atherosclerosis. Besides high LDL, elevated TG contributes to arterial wall thickening (arteriosclerosis), increasing the risk of stroke, myocardial infarction, and cardiovascular events [35, 36]. Hypertriglyceridemia is a significant independent risk factor for CVD, with its pathogenic mechanism involving complex pathophysiological interactions. Although TG themselves do not directly induce atherosclerosis, triglyceride‐rich lipoprotein (TRL) remnants serve as direct drivers of atherosclerotic progression. Due to their small size, TRLs easily penetrate the vascular endothelium and accumulate within the arterial wall, where they are engulfed by macrophages, forming foam cells—a hallmark of early atherosclerotic plaque formation [37, 38]. Once oxidized within the vessel wall, TRLs activate inflammatory pathways such as nuclear factor‐*κ*B, promoting monocyte adhesion and accelerating plaque progression [39]. Large population‐based studies, such as the Copenhagen General Population Study, have confirmed that elevated nonfasting TG levels are independently associated with an increased risk of myocardial infarction [40]. Furthermore, HTG exacerbates vascular injury through chronic inflammation and oxidative stress. Elevated TG levels activate Toll‐like receptor 4 (TLR4) and the NLRP3 inflammasome, leading to the release of pro‐inflammatory cytokines such as interleukin‐1*β* (IL‐1*β*) and IL‐6, thereby creating a proatherogenic microenvironment [41]. Additionally, free fatty acids released during TG metabolism increase mitochondrial reactive oxygen species production, resulting in endothelial oxidative stress and dysfunction [42, 43]. Furthermore, elevated AIP has been found to be closely related to insulin resistance. Dysregulation of glucose–lipid metabolism affects atherosclerosis and cardiovascular outcomes through the endogenous cGAS‐STING signaling pathway [44, 45]. Studies suggest that inhibiting the cGAS‐STING pathway significantly improves the progression of metabolic CVDs and prolongs survival in experimental animals [45]. Recent findings also suggest that AIP dysregulation may be associated with gut microbiota imbalance, exacerbating systemic lipid metabolism disorders through bile acid metabolism disturbances [46]. This suggests that the gut microbiota–metabolic axis may be a potential mechanism linking AIP and CVD. These mechanisms indicate that AIP is not only a biomarker of lipid metabolic dysregulation but also a crucial mediator connecting metabolic abnormalities and vascular injury. Although our study provides new insights into the relationship between AIP and CVD, further basic and clinical research is required to validate the impact of AIP on CVD.

Our study identified a significant U‐shaped association between AIP and the risk of CHF, suggesting that both excessively high and low AIP levels increase the risk of CHF. Low AIP levels may reflect excessively elevated HDL‐C, which may be associated with impaired cholesterol reverse transport and reduced vascular protection [47, 48]. In our study, when AIP > 0.22, higher AIP was significantly associated with increased CVD risk. Extremely high AIP suggests severe lipid metabolic imbalance, potentially causing direct myocardial damage through lipotoxicity, whereas moderate AIP may reflect a “compensatory period” of metabolic stress [49]. Several studies on lipids and CVDs have reported U‐shaped associations between non‐HDL cholesterol and all‐cause and cardiovascular mortality in the general population [50]. Similarly, a U‐shaped relationship was observed between the non‐HDL‐C/HDL‐C ratio and CVD in obese populations [51]. A nationwide population‐based study also demonstrated a U‐shaped association between AIP and CVD‐specific mortality. Although research focuses may differ, our findings are consistent with previous studies, suggesting that the relationship between AIP and CVD is not simply linear [15]. This U‐shaped association suggests that clinical practice should aim to maintain AIP within an optimal range, and future studies should further validate this phenomenon and explore its molecular mechanisms.

Our observed U‐shaped association between AIP and prevalent CHF with sharply higher odds above an inflection point aligns with the notion that extreme alterations in lipoprotein metabolism may reflect distinct pathophysiologic states (e.g., advanced cachexia or severe metabolic disturbance) that have nonlinear relations with CHF prevalence; similar complexity has been observed in other cohorts and warrants prospective validation [52]. Several mechanisms likely explain why associations between AIP and cardiovascular outcomes differ by subtype and age. Atherothrombotic diseases (e.g., coronary heart disease and myocardial infarction) are directly linked to dyslipidemia and the atherogenic processes described above, so a stronger and more linear positive association with AIP is biologically plausible [52]. By contrast, heart failure is etiologically heterogeneous, ischemic, hypertensive, valvular, and nonischemic cardiomyopathies contribute to CHF prevalence, and the relation between lipids and heart failure may therefore be nonlinear or modified by comorbidities and treatment [53]. This could contribute to U‐shaped or null associations seen for CHF in some reports.

Older adults often have multiple chronic diseases (e.g., chronic kidney disease and hypothyroidism), which may mediate CVD risk through nonlipid pathways (inflammation and vascular calcification), diminishing AIP′s independent predictive value [54, 55]. In addition, older adults are more likely to use statins and other preventative treatments to alter their lipid profile, and survival bias also weakens the cross‐sectional association. Younger adults with elevated AIP more often exhibit untreated atherogenic dyslipidemia and metabolic syndrome that strongly predispose to premature atherosclerosis. Several recent observational analyses have reported stronger AIP‐CVD associations in younger subgroups, consistent with our age‐stratified results. Moreover, in older populations, HDL′s anti‐inflammatory function declines with age, and LDL particles tend to become larger and more inert, which may offset the atherogenic effects of AIP [56, 57]. Since our findings are limited, future research should further explore how age modulates the pathological effects of AIP (metabolic reprogramming) and develop dynamic risk assessment models to better interpret this interaction.

Our study introduces several novel contributions to the existing literature. First, we systematically evaluated the relationship between the AIP and various CVD subtypes by employing a dual analytical framework–quartile‐based categorization and continuous variable modeling. This approach mitigated the limitations associated with relying on a single analytical method, commonly observed in prior studies. Second, our investigation was to compare the predictive capacity of AIP across different CVD subtypes, elucidating the specificity of its pathological mechanisms and offering a theoretical foundation for precision‐based risk stratification. Third, we were to identify a U‐shaped nonlinear relationship between AIP and CHF, with a threshold identified at AIP = 0.22, underscoring the need for cautious clinical intervention to prevent adverse outcomes associated with extreme AIP levels. Finally, we demonstrated that age significantly modified the association between AIP and CVD, highlighting that middle‐aged populations may derive greater benefit from AIP‐guided interventions, whereas older adults may require a more multidimensional risk assessment strategy.

Despite these strengths, our study has some inherent limitations. First, the cross‐sectional nature of the NHANES dataset precludes causal inference, limiting our ability to establish temporal relationships between AIP and CVD. Future research should incorporate longitudinal cohort designs or prospective studies to clarify causality. Second, although NHANES employs a complex multistage probability sampling design to ensure national representativeness, potential biases such as nonresponse and survivor bias may still affect the validity of the findings. Third, although we adjusted for numerous confounding factors, residual confounding from unmeasured variables, such as genetic predisposition and environmental exposures, may persist and influence the observed associations between AIP and CVD outcomes. In addition, we note that CVD outcomes in this analysis were defined by participants′ self‐report of ever having been told by a clinician that they had CHF, coronary heart disease, angina, heart attack, or stroke. As such, these outcomes reflect prevalent (lifetime) diagnoses rather than incident events and are susceptible to information bias, including recall error and differential ascertainment due to variation in healthcare access and utilization. Individuals who more frequently access healthcare or participate in routine screening are more likely to have conditions diagnosed and therefore more likely to report a prior diagnosis in survey data. If healthcare access and frequency of medical contact are associated with AIP, differential outcome misclassification could bias the estimated associations either toward or away from the null, depending on the direction of the association between access and AIP. By contrast, if misclassification of self‐reported CVD is nondifferential with respect to AIP, conventional measurement theory suggests that associations are most likely attenuated toward the null. In our multivariable models we adjusted for sociodemographic factors that partially proxy healthcare access (e.g., education level and family income‐to‐poverty ratio) to reduce confounding due to differential diagnosis. Nevertheless, residual bias from unmeasured dimensions of access (such as insurance coverage, regular source of care, or frequency of health checks) may remain. Where possible, future work should (i) validate self‐reported outcomes against medical records; (ii) examine incident events in prospective cohorts; or (iii) include direct measures of healthcare access/utilization as covariates or stratification variables to better assess and mitigate differential ascertainment.

## 5. Conclusion

Our study confirms that elevated AIP is significantly associated with increased risks of overall CVD, coronary heart disease, and heart attack, with a stronger impact observed in younger populations (< 55 years). A U‐shaped nonlinear relationship was observed between AIP and CHF, with a rapid increase in risk when AIP exceeded 0.22. These findings support the role of AIP as an important biomarker for CVD risk stratification and underscore the need for tailored intervention strategies targeting different CVD subtypes and age groups.

## Conflicts of Interest

The authors declare no conflicts of interest.

## Author Contributions

All authors contributed to the study conception and design. Y.H. and X.C. wrote the original manuscript. Q.W. and Y.L. performed the data analysis. W.X. reviewed and edited the manuscript.

## Funding

This study was supported by the Research on the Impact of Electromagnetic Interference on the Accuracy of Wearable Long‐Term Electrocardiogram Data (IEQRKL2408) and the Application Value of Regional Remote Electrocardiogram Network Construction in Improving the Precise Diagnosis and Treatment of Cardiovascular Diseases in Primary Hospitals (WHWJ2023y016).

## Data Availability

The data that support the findings of this study are openly available in the NHANES database at https://www.cdc.gov/nchs/nhanes/index.html.
